# Redox-Gated Optical Modulation of Coumarin-Triphenyliminophosphorane Fluorophores

**DOI:** 10.1021/acsphyschemau.4c00082

**Published:** 2024-11-04

**Authors:** Wei-Chu Huang, Yi-Yin Lu, Shiao-Chen Huang, Tai-Chung Lo, Shun-Yuan Luo, Wei-Hong Huang, Chih-Wei Luo, Vincent K.-S. Hsiao, Chih-Chien Chu

**Affiliations:** a Department of Medical Applied Chemistry, Chung Shan Medical University, Taichung 402, Taiwan; b Department of Chemistry, National Chung Hsing University, Taichung 403, Taiwan; c Department of Electrophysics, National Yang Ming Chiao Tung University, Hsinchu 300, Taiwan; d Department of Applied Materials and Optoelectronic Engineering, National Chi-Nan University, Puli 545, Taiwan; e Department of Medical Education, Chung Shan Medical University Hospital, Taichung 402, Taiwan

**Keywords:** coumarins, iminophosphorane, redox-responsive, radical emissive, two-photon excited fluorescence

## Abstract

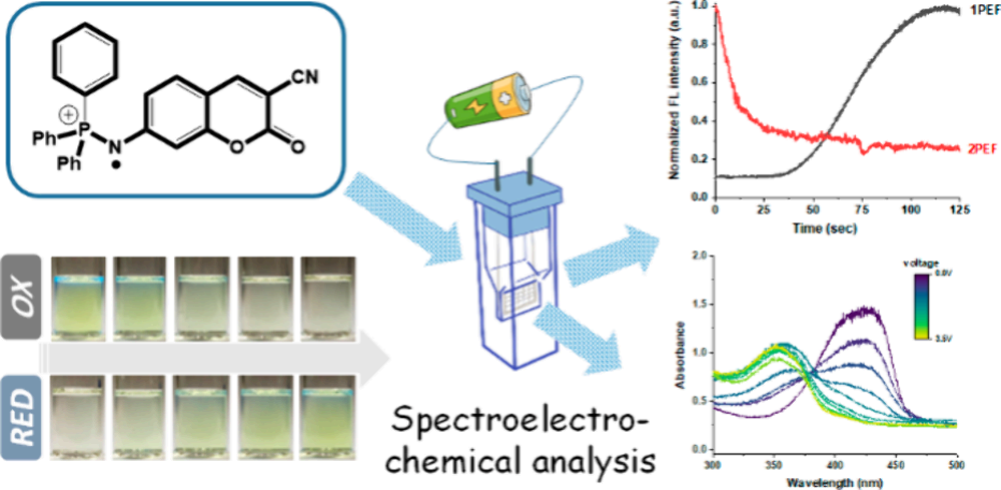

Novel coumarin-triphenyliminophosphorane (TPIPP) fluorophores, synthesized via a nonhydrolytic Staudinger reaction, exhibit remarkable redox-responsive optical properties. Upon chemical and electrochemical oxidation, these compounds display a hypsochromic shift in absorption from 430 to 350 nm, accompanied by up to 11-fold fluorescence enhancement under 405 nm excitation. The fluorescence switching occurs at an electrochemical oxidation potential of approximately +2.0 V. This enhanced one-photon excited fluorescence is attributed to an emissive radical effect, stemming from in situ generated radical cations at the polarizable iminophosphorane (P=N) bond. The radical formation was confirmed by trapping experiments using tetracyanoquinodimethane, which produced characteristic absorption of radical anions around 850 nm, and by electron spin resonance studies using 5,5-dimethyl-1-pyrroline *N*-oxide as a spin trap. Conversely, two-photon excited fluorescence under 800 nm pulsed laser excitation decreases upon oxidation, likely due to reduced two-photon absorption resulting from altered π-conjugation. This work demonstrates that external redox modulation can induce significant changes in absorption profiles and enable switching between enhanced one-photon and diminished two-photon excited fluorescence, highlighting the potential of leveraging the controllable radical character of the P=N bond in designing redox-responsive fluorophores.

## 1 Introduction

The pentavalent phosphoryl group, denoted as P=E with a formal double bond between the phosphorus center and an appropriate substituent (e.g., E = O, S, and Se), exhibits a strongly polarized bond feature.^1−4^ Besides common oxygen family substituents, the iminophosphorane (IPP) group with a highly polarized P=N bond has also garnered significant interest for designing “superbase” organocatalysts in asymmetric synthesis in the past decade.^5^ The superbasic nature of the IPP group can be attributed to the resonance contribution of the aza-ylide (P^+^-N^–^) form; this leads to p*K*_BH_^+^ values comparable to those of guanidine and amidine, which depend on the choice of phosphine ligands. Furthermore, the superbasic IPP moiety can be generated in situ by combining functional organoazides with commercial phosphine ligands via the Staudinger reaction.^6,7^ This enables late-stage tuning to the Brønsted basicity of the IPP-containing catalysts. One major concern with the IPP group is its stability in an aqueous reaction environment because the nucleophilic attack of water molecules leads to hydrolysis of the P=N bond to yield an amine functionality, expressed as the Staudinger reduction.^8^

Recently, the concept of a “nonhydrolytic” Staudinger reaction (NSR) has attracted much attention in the field of biorthogonal chemistry and has been considered an efficient “click type” strategy for material synthesis.^9−12^ The NSR involves the use of an electron-deficient aromatic azide with the introduction of electronegative fluorine or chlorine substituents, resulting in stabilization of the IPP group through an inductive effect. Our previous work also demonstrated that the NSR strategy can be used to create new fluorophores of coumarin-triphenyl-iminophosphorane (TPIPP), as the IPP group can be stabilized by the electron-withdrawing 2-pyranone ring and CN group in the coumarin structure through mesomeric effects.^13,14^ Furthermore, introducing an external CN group at the C-3 position of the coumarin ring favors the formation of a para-quinoidal resonance structure in the direction of the intramolecular charge transfer (ICT) state under photoexcitation. These novel coumarin fluorophores exhibit a remarkable two-photon absorption (2PA) behavior upon irradiating a femtosecond (fs) pulsed laser with a near-infrared (NIR) wavelength. Leito and co-workers also synthesized a series of coumarin-TPIPP derivatives as a lipophilic fluorescence indicator and later found that these fluorophores exhibit an interesting acidity-dependent optical response.^15,16^ Changing the environment acidity leads to a profound change in 2PA profiles due to chromophores’ π-conjugation switching between neutral and protonated P=N bond states. This fine manipulation is definitely based on the superbasic character of the IPP group and highlights the sensitivity of coumarin-TPIPP fluorophores to external stimuli.

Because of the strongly polarizing bond feature, it has been reported that the R_3_P=N group (R = aryl) also undergoes a reversible one-electron oxidation, revealing that the IPP bond is redox-active.^17−19^ The generation of the radical cation intermediates can be further stabilized through charge delocalization by the phosphine ligand. However, there has rarely been a discussion in the literature on whether redox stimulation produces corresponding changes in optical properties of the IPP group-containing chromophores. Accordingly, in addition to the pH response, we further investigated the redox-responsive property of coumarin-TPIPP derivatives through chemical and electrochemical methods. The absorption behavior of these derivatives can be tuned between oxidative and reductive treatments. Moreover, the fluorescence exhibits significant intensity changes in the oxidation and reduction states, enabling fluorescence switching upon external redox stimulation.

## 2 Results and Discussion

**Figure 1:**
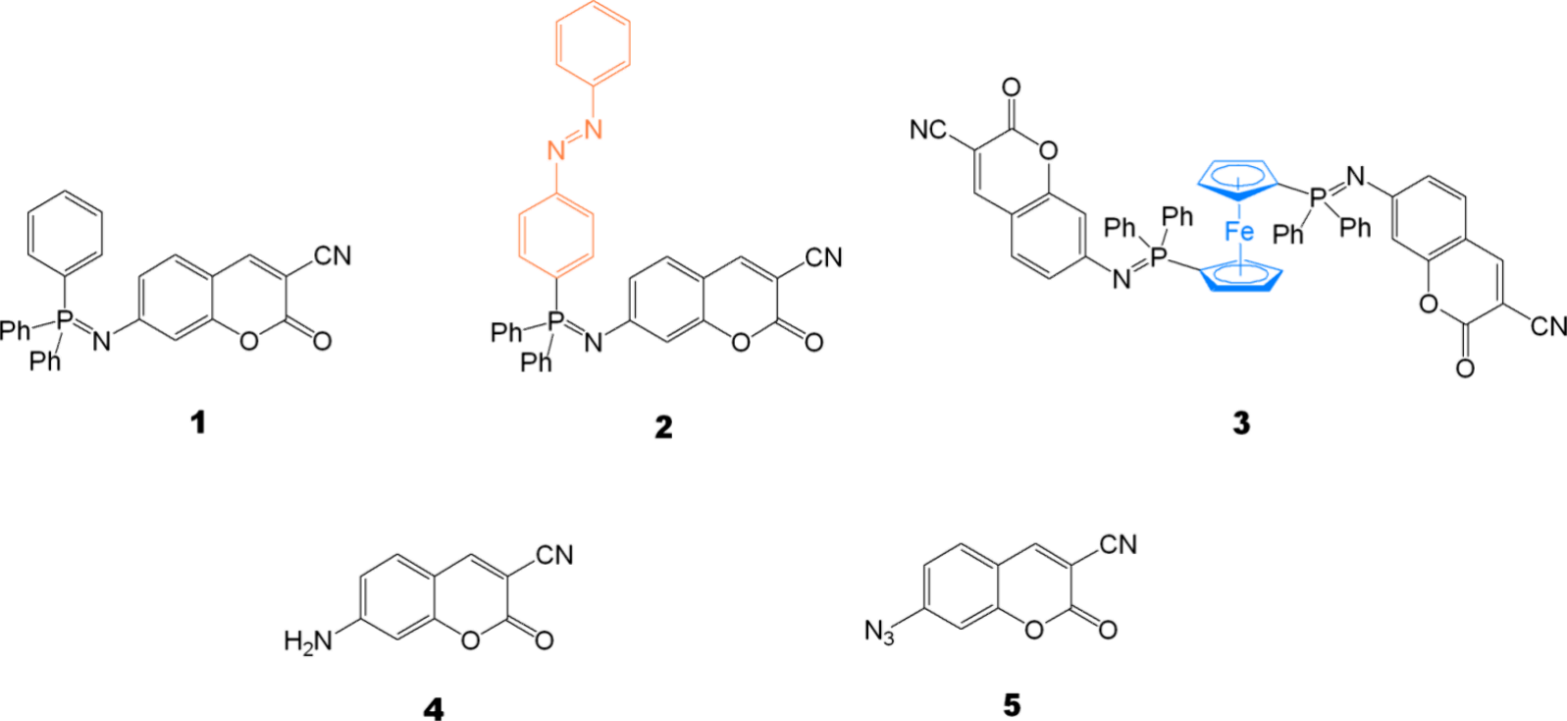
Chemical structures of the coumarin-triphenyliminophosphorane (TPIPP) derivatives **1**–**3** and 7-amino- and 7-azido-3-cyanocoumarins **4** and **5**.

Figure 1 shows the chemical structures of the coumarin-TPIPP compounds **1**–**3**, which were readily synthesized by the NSR of 7-azido-3-cyanocoumarin (**4**) and three phosphine ligands, including triphenylphosphine (PPh_3_), azobenzene- (Az), and ferrocene (Fc)-containing phosphanes.^20,21^Table 1 summarizes the photophysical data for the coumarin-TPIPP with the π–π* absorption peaks at approximately 430 nm. In particular, the latter two ligands can effectively quench or reduce the coumarin-TPIPP compounds’ fluorescence through electron or energy transfer.^22^ Moreover, compound **3** possesses the highest molar extinction coefficient (ε) because one 1,1-bis(diphenyl-phosphino)-ferrocene (DPPF) was covalently linked with two 7-azido-3-cyanocoumarins.

**Table 1:** Photophysical Data of Coumarin-TPIPP Derivatives before and after Oxidation^a^

**compound**	λ_max_ (nm)	ε_max_ (10^4^ M^–1^cm^–1^)	λ_max_^fl^ (nm)^c^	Φ_f_^d^
**1**	426	4.39	460	1.33
**2**	425	3.04	450	ND
**3**	430	7.13	460	0.18
**4**	397	1.53	433	1.59
**5**	354	3.19	432	0.36
**1-ox**^b^	353	2.67	408	3.32
**2-ox**^b^	339	2.21	430	0.03
**3-ox**^b^	354	5.66	409	2.05

a The reaction was performed in anhydrous tetrahydrofuran (THF).

b Oxidized by iron(III) perchlorate.

c Excitation wavelength is absorption maximum.

d Relative quantum yields calibrated by coumarin 337, as reported in ref (14) (Φ_f_ = 0.94 in acetone).

**Figure 2:**
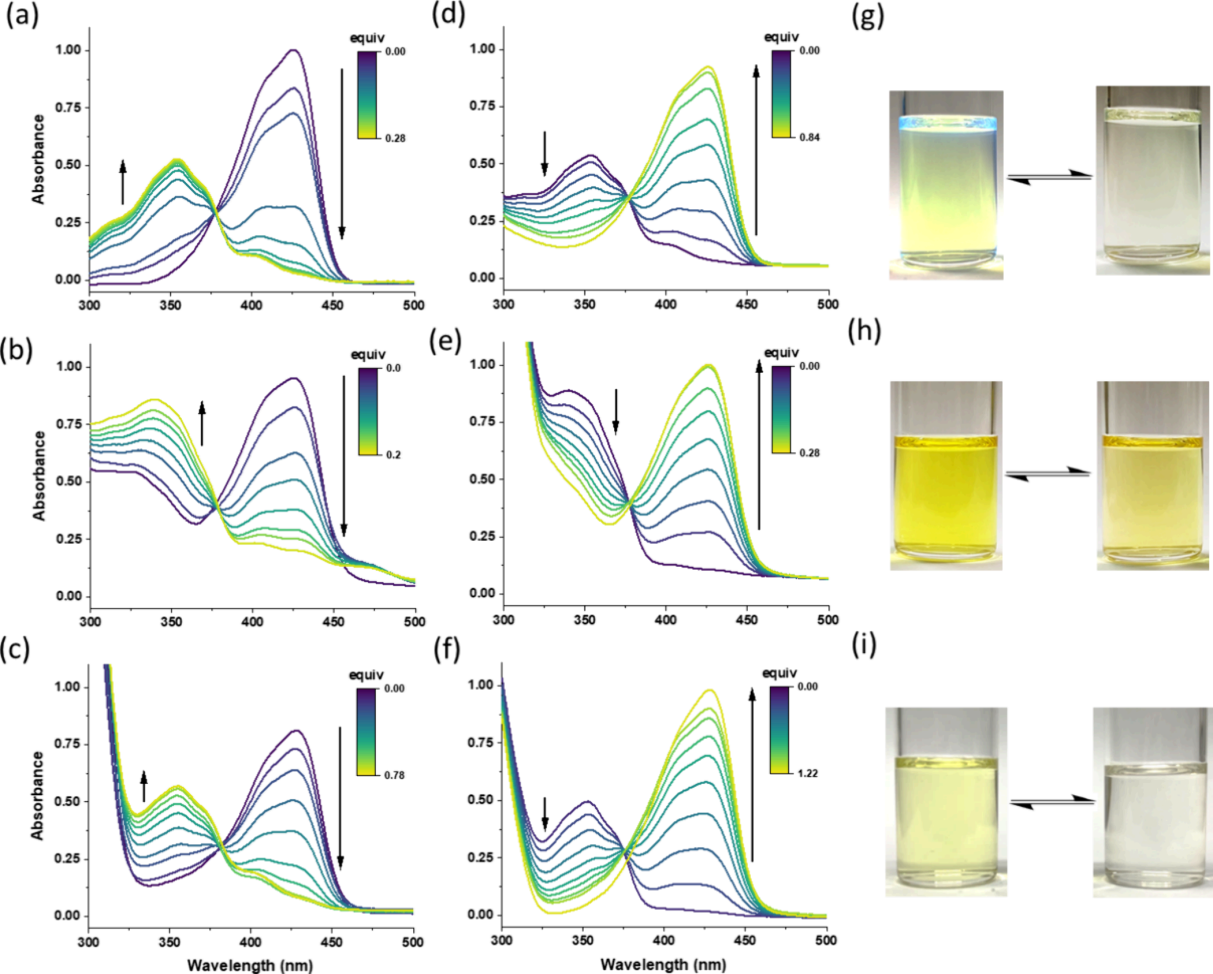
UV–vis absorption spectra of (a–c) coumarin-TPIPP compounds **1**–**3** undergoing oxidation by Fe(ClO_4_)_3_ and (d–f) subsequent reduction by LAS. The spectral lines evolve from purple to green with increasing equivalents of Fe(ClO_4_)_3_ and LAS, with arrows indicating the direction of spectral changes. Photos showing the color change of compounds (g) **1**, (h) **2**, and (i) **3** during redox cycling. [**1**–**3**] = 2 × 10^–5^ M in THF.

To perform the chemical oxidation–reduction cycles, iron(III) perchlorate (Fe(ClO_4_)_3_) and sodium l-ascorbate (LAS) were used as the oxidant and reductant, respectively.^21^Figure 2a–c shows the change in UV–vis absorption profiles after the oxidative treatment of compounds **1**–**3**. The absorption peak at 430 nm was gradually decreased by increasing the amount of the iron(III) oxidizing reagent added, and the blue-shifted absorption peak at 350 nm was increased to a plateau simultaneously. The ε values of the oxidized compounds decrease by approximately 20–40% (Table 1). Subsequently, as shown in Figure 2d–f, the absorption spectra can be recovered by gradually adding LAS to the oxidized solutions. The reversible change in the color of compounds **1**–**3** solutions is shown in Figure 2g–i, respectively. It can be found that the oxidized solutions became decolorized, which is related to the shift of the absorption spectra from the visible to UV-light wavelength.

It is noticed that three coumarin-TPIPP compounds exhibit a similar change in the absorption spectra, regardless of the phosphine ligands used. Furthermore, both 7-azido and 7-amino-3-cyanocoumarins (**4** and **5** in Figure 1) exhibit no spectral change under oxidative treatment. Therefore, we assumed that the redox-active behavior should be attributed to the P=N bond feature. Leito and co-workers have reported that the blue-shifted absorption pattern of the acidified coumarin-TPIPP compound results from the protonated nitrogen atom in the P=N bond.^16^ As a result, the C7-derived TPIPP group no longer acts as an electron-donating substituent to form an electronic “push–pull” coumarin structure with red-shifted absorption wavelengths.^23^ In our system shown in Figure 2a–f, the fact that isosbestic points at approximately 380 nm could be observed during the titration process also supports a simple chemical transformation between two species with different coumarin-derived structures and π-conjugation frameworks.^24^ According to previous studies, electrochemical oxidation (ECO) of the P=N bond will generate the radical cation intermediate, which can be further stabilized through charge delocalization by the phosphine ligand. We assumed that the formation of radical cations at the P=N bond for the oxidized compounds may be crucial in diminishing the electron-donating ability of the TPIPP group and changing the π-conjugation structure. The spectra show blue-shifted absorption patterns centered at 350 nm, which is closely matched with the 3-cyanocoumarin chromophore reported in the literature.^25,26^

**Figure 3:**
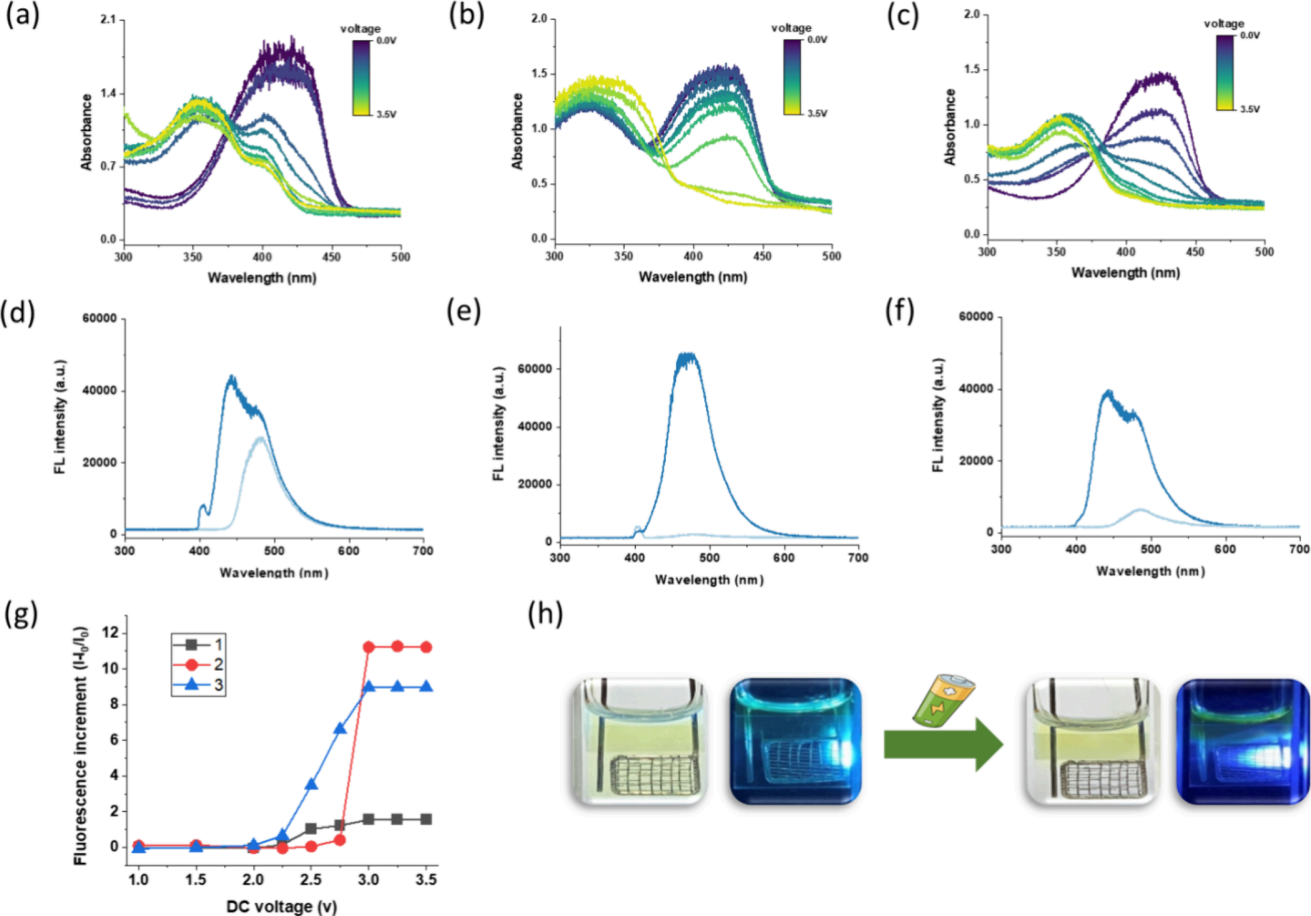
Spectroelectrochemical (SEC) analysis: UV–vis absorption spectra of (a) **1**, (b) **2**, and (c) **3** during electrochemical oxidation using a DC power supply (0 to +3.5 V). The spectral lines evolve from purple to green with increasing applied voltages. Fluorescence spectra of (d) **1**, (e) **2**, and (f) **3** recorded before and after electrochemical oxidation at +3.5 V under 405 nm excitation. (g) Increment ratio of fluorescence intensity of **1**–**3** versus increasing DC voltages. (h) Photo of the emissive oxidized solution of **1** around the gauze electrode using a 3 V battery. [**1**–**3**] = 2.5 × 10^–4^ M, [TBAPF_6_] = 2.5 × 10^–2^ M in THF.

The ECO process for the coumarin-TPIPP compounds was then driven by a power supply equipped with adjustable applied DC voltages, and then, spectroelectrochemical (SEC) analysis was performed on a home-built apparatus. The Pt gauze electrode was used as the working electrode in the presence of tetrabutylammonium hexafluorophosphoate (TBAPF_6_) as the supporting electrolytes, and the absorption and emission spectroscopic signals were collected by fiber optics and in real time recorded by a CCD-array spectrometer (Supporting Information). Figure 3a–c shows the blue-shifted absorption patterns of the compounds **1**–**3**, respectively, after the ECO treatment; the absorption peak at 430 nm gradually decreased as the applied voltage continuously increased to +3.5 V; meanwhile, the blue-shifted absorption peak at approximately 350 nm increased simultaneously, which is consistent with the chemical titration experiments (Figure 2a–c). The result suggests that the oxidized species continuously accumulated in the solution by increasing the applied voltage toward positive bias. Furthermore, the change in fluorescence spectra of compounds **1**–**3** was recorded under 405 nm laser excitation. Interestingly, the ECO process results in fluorescence enhancement with slightly blue-shifted wavelengths under +3.5 V, as shown in Figure 3d–f, respectively. In particular, compounds **2** and **3** exhibit a significant ECO-triggered fluorescence “turn-on” effect because the intrinsic emission of the coumarin-TPIPP was quenched or greatly reduced by Az and Fc-derived phosphine ligands.

As shown in Figure 3g, the increment ratios of fluorescence intensity for compounds **1**–**3** were further analyzed under increasing voltages up to +3.5 V; each voltage persists for 150 s to attain equilibrium, after which the fluorescence spectra were recorded. It is found that the threshold gate voltage for fluorescence increment of **1** and **3** was approximately +2.0 V, and the fluorescence intensity increases rapidly above +2.25 V; the threshold voltage for **2** was slightly higher at +2.5 V, and the fluorescence suddenly increased at +2.75 V. Eventually, the redox-stimulated optical response can also be carried out using a simple 3 V dry cell battery. Figure 3h shows the oxidized compound **1** around the gauze electrode. The solution became decolorized and emitted stronger blue fluorescence under a “battery-driven” ECO process.

**Figure 4:**
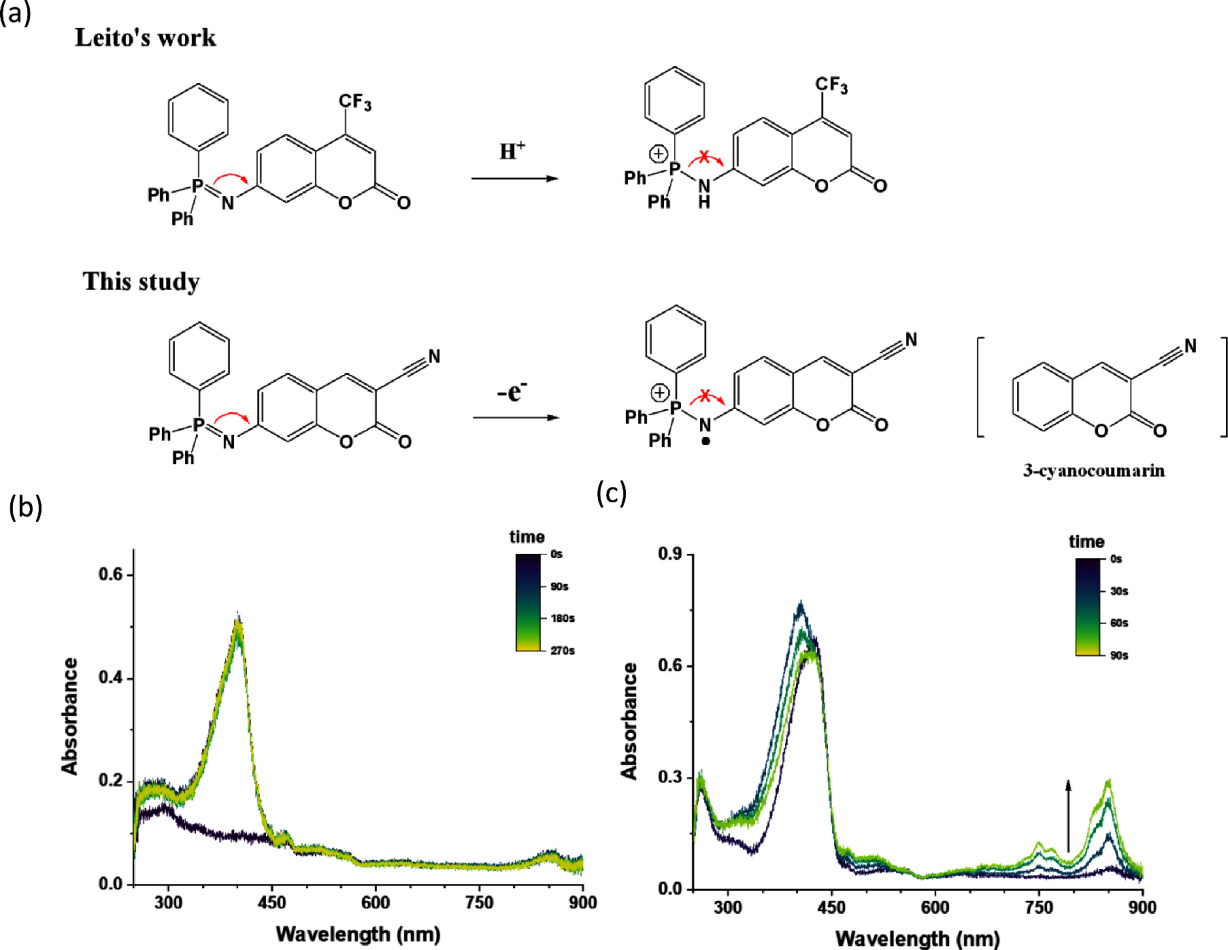
(a) Proposed mechanisms of electrochemical oxidation (ECO) of the P=N bond leading to a radical cation versus protonation of P=N to a phosphonium cation. UV–vis absorption of (b) blank and (c) electrochemically oxidized **1** after addition of TCNQ as the radical trapping reagent, confirming formation of the TCNQ radical anion at the NIR region. [**1**] = 1.25 × 10^–4^ M, [TBAPF_6_] = 1.25 × 10^–2^ M, [TCNQ] = 1.25 × 10^–3^ M in THF. The ECO process was driven by a 3 V battery.

We further analyzed relative fluorescence quantum yields (Φ_f_) using coumarin 337 as a calibrating standard (Table 1).^27^ The compounds **1** and **3** exhibit an approximately 2.5- and 11-fold increase in quantum yields after chemical oxidation. The fluorescence increments of compounds **1** and **3** under the ECO process were found to be 1.6- and 9-fold, which is in agreement with the increment of quantum yields after chemical oxidation. Notably, the Az group is a highly effective fluorescence quencher so that the Φ_f_ of compound **2** is too low to be determined until oxidative treatment was performed. The increase in fluorescence of the coumarin-TPIPP compounds after oxidative treatment is opposite to the decrease in fluorescence of the compounds after acidic treatment reported by Leito and co-workers^16^ ; as shown in Figure 4a, the fluorescence quantum yields of the acidified P=N bond decreased dramatically by more than 90%. In our system, oxidation of the P=N bond producing the radical cation may cause the TPIPP group to lose electron-donating ability. Therefore, compared to the original coumarin-TPIPP, these oxidized species share a comparable chromophore structure and absorption pattern with 3-cyanocoumarin, which lacks a C7-substituted electron-donating group. This is also consistent with the coumarin analogue produced by the acidified coumarin-TPIPP compounds. However, the reported Φ_f_ value of 3-cyanocoumarin is extremely low at approximately 0.006–0.02, indicating that 3-cyanocoumarin itself is not highly fluorescent.^25,26^ Therefore, the formation of 3-cyanocoumarin synthons only accounts for the blue-shifted absorption profiles but does not explain the fluorescence enhancement observed for the oxidized coumarin-TPIPP derivatives. Alternatively, radical formation after oxidative treatment may be crucial to fluorescence enhancement. To confirm the radical formation after the oxidation, an excellent electron acceptor related to tetracyanoquinodimethane (TCNQ) was added to the ECO-treated solution of **1**. As shown in Figure 4b, the blank solution containing only the supporting electrolytes for transducing electrochemical reactions has very weak absorption peaks at NIR wavelengths (700–900 nm) upon TCNQ addition; in contrast, solution **1** has significant absorption peaks at the NIR region (Figure 4c) and turns into a deep green color. This transformation reveals the characteristic absorption of the radical anion of TCNQ, which may capture an electron from oxidized **1**. The TCNQ assay method evidently suggests the radical formation of coumarin-TPIPP after the ECO process.

**Figure 5:**
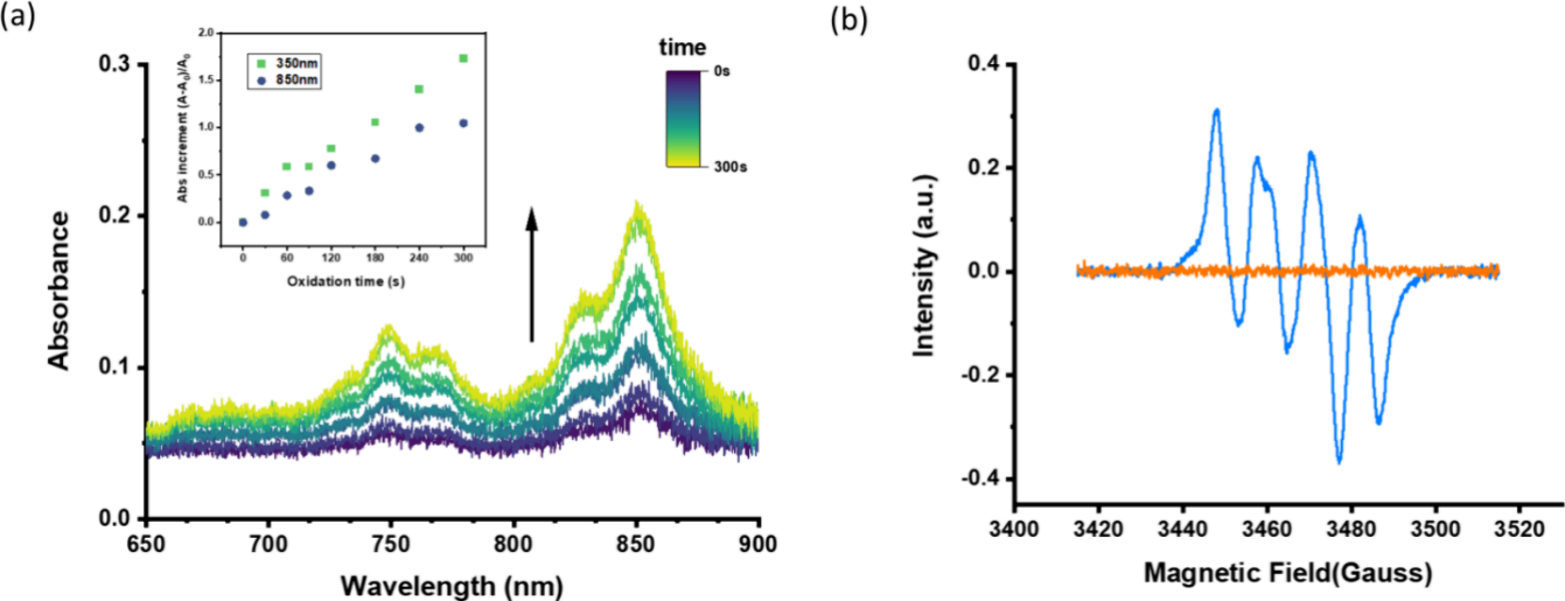
(a) UV–vis absorption spectra of **1** zoomed in the NIR region upon increasing oxidation times up to 300 s followed by TCNQ addition. The inset shows the absorbance increment at 350 and 850 nm versus oxidation time. The spectral lines evolve from purple to green with increasing oxidation time. (b) ESR spectra of THF solutions: electrolyte alone (red) and with **1** (blue) after +3.0 V application for 300 s. The emerging signal at *g* ≈ 2.003 indicates organic radical formation upon oxidation.

We further analyzed the correlation between the ECO-treating time for compound **1** and the generation of the radical anion of TCNQ by recording the real-time absorption spectra (Supporting Information). Before the addition of TCNQ, the oxidation time of compound **1** was set to 30, 60, 90, 120, 180, 240, and 300 s. It was found that the longer oxidation time resulted in a more pronounced absorption peak at 350 nm, indicating that more oxidized **1** was formed. Subsequently, after the addition of TCNQ and monitoring for up to 180 s, it is observable that the absorption peaks in the NIR region progressively increased. As shown in Figure 5a, upon zooming in the 700–900 nm wavelength range, the production of TCNQ radical anions gradually reaches the maximum level as the oxidation time increases to 300 s. Upon further analysis shown in the inset of Figure 5a, it is evident that both the absorbance increments in the 350 and 850 nm range increased proportionally with the oxidation time. The result indicates that an increase in the oxidation time leads to a greater production of radical species in the oxidized coumarin-TPIPP. Consequently, a greater accumulation of TCNQ radical anions occurs within the system through an electron transfer process.

To further corroborate the formation of organic radicals upon electrochemical oxidation, we conducted electron spin resonance (ESR) spectroscopy measurements. Figure 5b shows the ESR spectra of the electrolyte solution with and without compound **1**, both after applying a 3 V potential. The electrolyte-only sample (red line) exhibits no discernible ESR signal, indicating that oxidation of the solvent or supporting electrolyte does not produce stable paramagnetic species. However, upon addition of compound **1** to the system (blue line), a clear ESR signal emerges under the same oxidative conditions, characterized by a single, relatively broad peak approximately centered at *g* ≈ 2.003. This g-factor value is typical of organic radicals. The appearance of the ESR signal only in the presence of compound **1** provides strong evidence for the generation of paramagnetic species during the oxidation process, specifically from our coumarin-TPIPP derivative. The broadness of the ESR peak suggests that the unpaired electron may be delocalized over the conjugated system of the coumarin-TPIPP structure, potentially involving both the coumarin core and the iminophosphorane moiety. This delocalization is consistent with our proposed mechanism of radical cation formation at the P=N bond and the subsequent electronic redistribution. The ESR results, in conjunction with our spectroscopic and chemical trapping experiments, provide compelling evidence of the formation of stable organic radicals upon electrochemical oxidation of coumarin-TPIPP compounds. These findings not only validate our proposed mechanism but also underscore the potential of these systems as redox-responsive paramagnetic probes.

Taking all data into account, we assumed that the increased fluorescence is likely attributed to the oxidative treatment itself acting on the P=N bond in coumarin-TPIPP structures. Based on the successful radical trapping experiment, we speculated that the fluorescence enhancing phenomenon results from the “spin doublet” effect, which often accounts for the unique luminescence property of organic radical molecules.^28,29^ Generally, radical molecules are considered as an open-shell molecule that has a doublet ground state with an unpaired electron; due to a spin-allowed doublet–doublet transition, the relaxation of the exciton from the lowest excited state to the ground state is highly efficient. Thus, this effect overcomes the problem of the forbidden transition from the triplet exciton to the ground state and leads to highly luminescent molecules. The fluorescence lifetime measurements for **1** before and after ECO treatment are 2.54 and 3.75 ns, respectively, which are typical for organic fluorophores.^30^ Additionally, based on the fact that the lifetime of compound **1** slightly increased after oxidation, we assumed that this is a significant feature in relation to the doublet–doublet (D_1_ →D_0_) transition of the open-shell system.^31^ This is because the nonradiative decay of intersystem crossing and internal conversion is faster for the singlet–singlet (S_1_ →S_0_) transition of the closed-shell system. Although the hypothesis requires further investigation, the coumarin-TPIPP derivatives should provide a pioneering example of in situ-generated emissive radical molecules under redox stimulations.^32,33^

**Figure 6:**
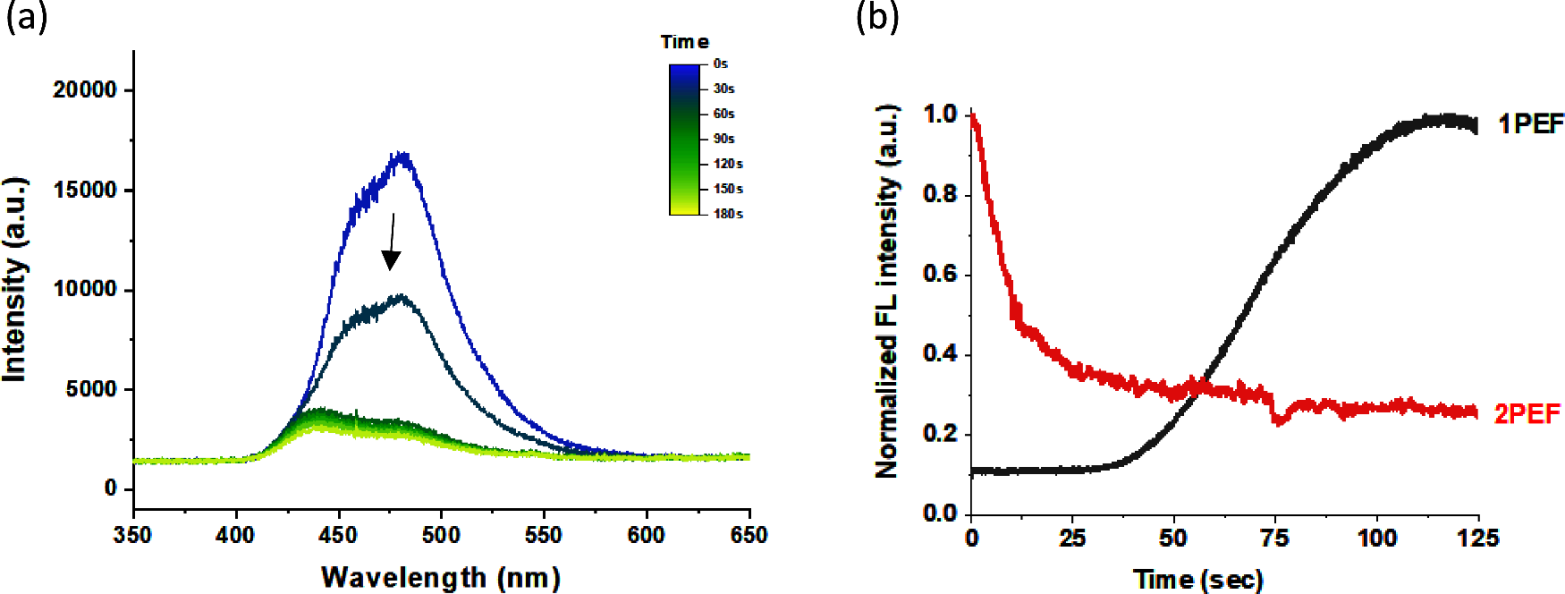
(a) SEC analysis showing evolution of two-photon excited fluorescence (2PEF) spectra of **1** with increasing oxidation time (0–180 s) under 800 nm fs-pulsed laser irradiation. (b) One-photon excited fluorescence (1PEF,405 nm) and 2PEF show opposite redox modulations of the fluorescence intensity. The electrochemical cell was driven by a 3 V battery. [**1**] = 2.5 × 10^–4^ M in THF.

The influence on the two-photon excited fluorescence (2PEF) under redox stimulation was eventually analyzed; as shown in Figure 6a, the 2PEF intensity of compound **1** gradually decreased under 800 nm fs pulsed laser irradiation, showing a reverse “turn-off” effect as compared with the fluorescence enhancement under one-photon 405 nm excitation (Figure 3d). The decrease in 2PEF is apparently due to the decrease in 2PA efficiency, suggesting that the coumarin-TPIPP has a relatively lower 2PA cross section at the oxidation state. A few report shows that the 2PA cross section greatly increased as the chromophore structure interconverted from a closed-shell dication dimer to open-shell diradical tetracation species.^34^ However, our result is consistent with Leito et al.’s work that the 2PA cross section of the coumarin-TPIPP compounds decrease by approximately 80% in the acidic environment.^16^ Because the TPIPP group is incapable of acting as an electron-donating substituent upon protonation or oxidation of the P=N bond, the electronic push–pull effect was reduced, and then, the 2PEF became weaker. Overall, as shown in Figure 6b, we successfully demonstrate that the external redox stimulation can produce real-time optical change of the coumarin-TPIPP compounds, particularly significant turn-on and turn-off fluorescence under one-photon and two-photon excitation (1PEF vs 2PEF), respectively.

## 3 Conclusions

In this work, we have demonstrated the redox-responsive optical properties of novel coumarin-TPIPP fluorophores synthesized via an efficient NSR protocol. Chemical and electrochemical oxidation treatments enabled significant changes in the absorption profiles, with a hypsochromic shift from 430 to 350 nm. Notably, we observed up to an 11-fold enhancement in one-photon excited fluorescence upon 405 nm excitation after oxidation, while the two-photon excited fluorescence under 800 nm pulsed laser irradiation was diminished. The spectroscopic changes were attributed to the formation of radical cations at the polarizable P=N bond, as confirmed by the successful trapping of radical species with tetracyanoquinodimethane. The fluorescence enhancement was proposed to originate from an emissive radical effect, enabling efficient doublet–doublet transitions in the open-shell oxidized species. This study pioneers the development of redox-stimuli-responsive coumarin compounds by leveraging the redox-active character of the iminophosphorane moiety. The ability to switch between enhanced one-photon and diminished two-photon excited fluorescence through external redox modulation presents opportunities for developing novel optical probes and fluorescence imaging applications. Further investigations into the photophysical properties and potential applications of these unique redox-active fluorophores are warranted.

## 4 Experimental Section

The detailed account for material synthesis and characterization is shown in the Supporting Information. The SEC measurement was performed on a home-built apparatus equipped with either a deuterium tungsten halogen light source (DT-Mini, Ocean Optics, USA) or 405 nm laser diode for absorption and fluorescence analysis, respectively; a CCD-array spectrometer (USB4000, Ocean Optics, USA) was used as the detector for analyzing the fiber-based optical signal. The SEC set equipped with a specific cuvette of optical path length of 1.0 mm and a Pt gauze working electrode was purchased from ALS Co., Ltd. (Japan). Either a battery or a DC power supply (E3631A, Agilent, USA) was used as the power source to perform the ECO process. To confirm the radical formation after the ECO process driven by a 3 V battery, the TCNQ was added to the ECO-treated solution to capture the formed radicals. The concentrations of the samples and TBAPF_6_ electrolyte are 2.5 × 10^–4^ M and 2.5 × 10^–2^ M in THF, respectively. The ECO-induced radical formation was analyzed by a TCNQ method. Before addition of the TCNQ, the oxidation time was set to 1, 30, 60, 90, 120, 180, 240, and 300 s followed by the addition of TCNQ and monitoring for up to 180 s for each ECO-treated solution. The concentrations of the samples, TBAPF_6_ electrolyte, and TCNQ are 1.25 × 10^–4^ M, 1.25 × 10^–2^ M, and 1.25 × 10^–3^ M in THF, respectively. X-band continuous wave ESR measurements were performed using an ER 4122 SHQE resonator on a home-built spectrometer consisting of a Bruker ER 049X microwave bridge with an EMX 114 microwave controller. The in situ electrochemical oxidation during ESR spectral acquisition was performed on a custom-built electrochemical cell equipped with a Pt wire as a working electrode. The sample solution containing compound **1**, TBAPF_6_ electrolyte, and 5,5-dimethyl-1-pyrroline *N*-oxide (DMPO) as a spin trapping agent in THF was then oxidized by applying a +3.0 V potential using a DC power supply (E3631A, Agilent, USA) during the ESR measurements. Fluorescence lifetimes were determined utilizing the time-correlated single photon counting technique. A digital converter (PicoQuant Time Harp 200), integrated into a computer system, measured the time delay between the excitation pulse and the detection of the first fluorescence photon. The fluorescence excitation source employed was a 375 nm pulsed diode laser emitting 80 ps pulses at a frequency of 40 MHz. These pulses were directed toward the sample through a focused lens, and the resulting fluorescence light was collected by a set of collimated lenses. Subsequently, the fluorescence was detected by a single-photon counting photomultiplier tube (PMT 957, Hamamatsu), which was equipped with an Olympus HR320 spectrometer. The fitting method was employed using the built-in exponential decay equation within the ORIGIN software. The 2PEF spectra in solution phase were measured by the experimental setup briefly described below: the laser source was based on a Solstice Ace amplifier (Spectra-Physics, USA), which delivers <120 fs pulses with a repetition rate of 2 kHz. The intensity of the incident beam was carefully controlled by the neutral density (ND) filter, and the beam was then divided by a 50/50 splitter before striking on the power meters. The excitation beam was focused on the SEC quartz cell, and the 2PEF was collected and induced by a fiber bundle into a CCD imaging spectrometer (USB4000, Ocean Optics, USA) for the spectra recording.

## Supporting Information Available

General information, synthesis of the azobenzene-derived phosphine ligand, general NSR procedure for compounds 1–3, spectroelectrochemical measurements and TCNQ analysis, and fluorescence life-time measurements, (PDF)
Real-time SEC video (MP4, MP4)
