# Supplementary material for: Redox-Gated Optical Modulation of Coumarin-Triphenyliminophosphorane Fluorophores
Source: ACS Phys Chem Au. 2024 Nov 4;5(1):92–100. doi: 10.1021/acsphyschemau.4c00082 (PMC11758267; doi:10.1021/acsphyschemau.4c00082)

Supporting Information

Redox-gated optical modulation of Coumarin-Triphenyliminophosphorane Fluorophores

Wei-Chu Huang,[a] Yi-Yin Lu,[a] Shiao-Chen Huang,[a] Tai-Chung Lo,[b] Shun-Yuan Luo,[b] Wei-Hong Huang,[c] Chih-Wei Luo,[c] Vincent, K.-S. Hsiao,[d] and Chih-Chien Chu,*[ae]

[a]Department of Medical Applied Chemistry, Chung Shan Medical University, Taichung 402, Taiwan
[b]Department of Chemistry, National Chung Hsing University, Taichung 403, Taiwan
[c]Department of Electrophysics, National Yang Ming Chiao Tung University, Hsinchu 300, Taiwan.
[d]Department of Applied Materials and Optoelectronic Engineering, National Chi-Nan University, Puli 545, Taiwan
[e]Department of Medical Education, Chung Shan Medical University Hospital, Taichung 402, Taiwan

*Corresponding Author: Prof. Dr. Chih-Chien Chu;

TEL: +886-4-36097617; e-mail: **jrchu@csmu.edu.tw**

1.1 General Information

The chemical reagents and organic solvents for materials synthesis were obtained as high-purity reagent-grade from commercial suppliers including Sigma-Aldrich and TCI chemicals and used without further purification. Compound **4** and **5** were synthesized following our published procedures.[1,2] ^{1}H (400 MHz) and ^{13}C (101 MHz) NMR spectra were recorded on a Varian Mercury Plus 400 MHz spectrometer at room temperature using $CDCl_3$, DMSO-d_6, as the solvents. Spectral processing (Fourier transform, peak assignment, and integration) was performed using MestReNova 6.2.1 software. High-resolution mass spectroscopy (HRMS) was performed on a JMS-T100LP 4G (JEOL) mass spectrometer equipped with the ESI source, detecting positive and negative ions. Typical measurement conditions are as follows: needle voltage: 2000 kV, orifice 1 voltage: 300 V, ring lens voltage: 10 V, spray temperature: 250°C. UV-Vis absorption spectra was performed on a Thermo Genesys 10S UV–Vis spectrometer. Fluorescence emission spectra was recorded on a Hitachi F-2700 spectrometer.

1.2 Synthesis of azobenzene-derived phosphine ligand

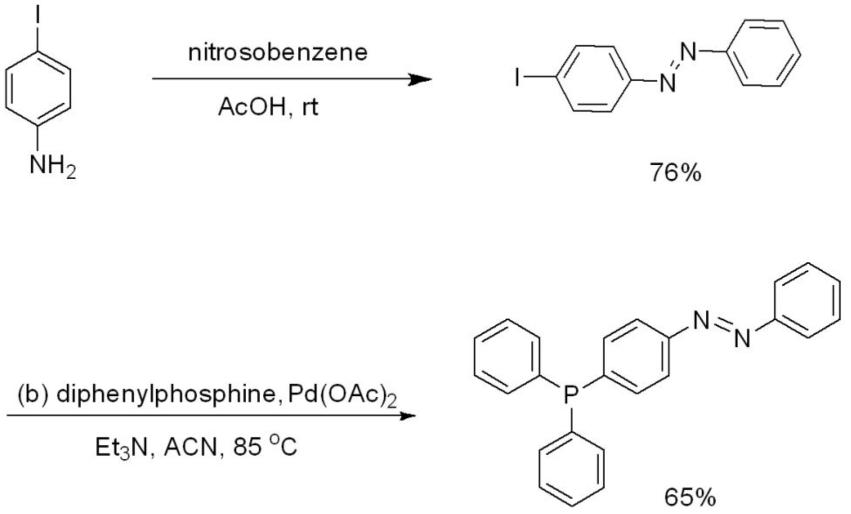

1.2.1 (*E*)-1-(4-Iodophenyl)-2-phenyldiazene: To a solution of 4-iodoaniline (1 g, 4.57 mmol) was mixed with nitrosobenzene (489 mg, 4.57 mmol) and acetic acid (20 mL). The reaction mixture was stirred at 25 °C for 4 hours. When the reaction finished, the mixture was poured into 10 mL of water and extraction with ethyl acetate (2×10 mL) the combined organics were washed with brine (5 mL), The organic layers were collected, dried over $MgSO_4$ and concentrated to dryness. The residue was purified by chromatography to afford desired product (532.0 mg, 76%) as orange solid. R_f 0.78 (EtOAc/Hex = 1/3); mp=97-100 °C; IR (KBr) ν 3055, 1585, 544 cm^{-1}; ^{1}H NMR (400 MHz, $CDCl_3$) δ 7.94 – 7.90 (m, 2H), 7.89 – 7.83 (m, 2H), 7.68 – 7.63 (m, 2H), 7.56 – 7.48 (m, 3H); ^{13}C NMR (101 MHz, $CDCl_3$) δ 152.4, 151.8, 138.3, 131.3, 129.1, 124.4, 122.9, 97.7; HRMS (ESI, M+H$^+$) calcd for

$C_{12}H_9IN_2$ 308.9888, found 308.9900.

1.2.2 (*E*)-1-(4-(diphenylphosphanyl)phenyl)-2-phenyldiazene: A solution of diphenylphosphine (187 μL, 1.07 mmol) in acetonitrile (8 mL), and a mixture of (*E*)-1-(4-Iodophenyl)-2-phenyldiazene (276 mg, 0.90 mmol) and palladium(II) acetate (6 mg, 0.027 mmol) were added to a flask. N,N-Diethylethanamine (125 μL, 0.90 mmol) was added to the solution, and the reaction mixture was stirred at 85 ºC for 1 hour. The dark orange mixture was cooled to room temperature and the solvent was removed. Orange solid of the pure product were obtained after recrystallization from ethanol to afford desired product (213.3 mg, 65%) as dark orange solid. R_f 0.71 (EtOAc/Hex = 1/4); mp=95-99 ºC; IR (KBr) ν 3068, 3052, 1584, 1480 cm^{-1}; ^{1}H NMR (400 MHz, $CDCl_3$) δ 7.93 – 7.89 (m, 2H), 7.86 (dd, *J* = 8.4, 1.3 Hz, 2H), 7.55 – 7.47 (m, 3H), 7.45 – 7.40 (m, 2H), 7.39 – 7.33 (m, 10H); ^{13}C NMR (101 MHz, $CDCl_3$) δ 152.6, 152.5, 141.3, 141.1, 136.6, 136.5, 134.2, 134.0, 133.9, 133.8, 131.1, 129.1, 128.9, 128.6, 128.6, 122.9, 122.7, 122.6; HRMS (ESI, M+H$^+$) calcd for $C_{24}H_{20}N_2P$ 367.1364, found 367.1364.

1.3 General NSR Procedure for compound 1-3

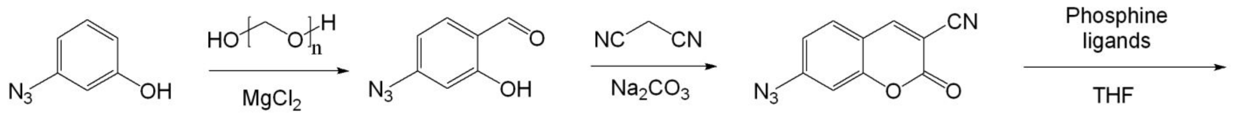

5

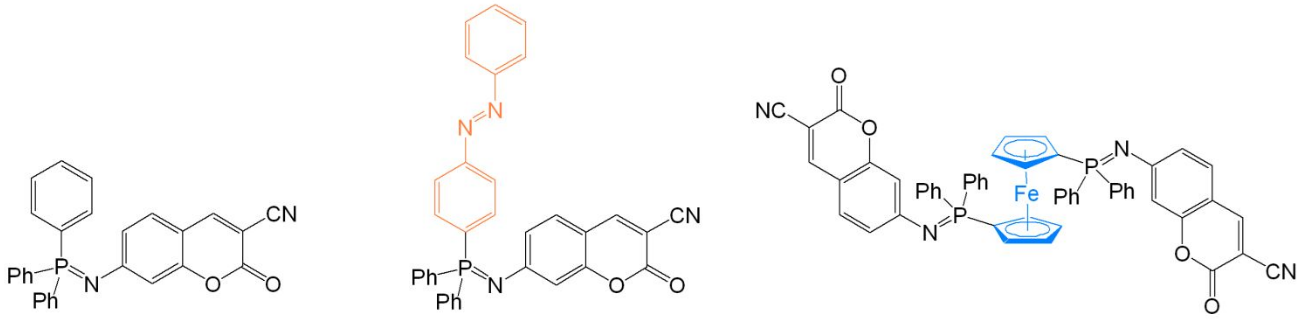

1 **2** **3**

7-azido-4-methylcoumarin (**5**, 20 mg, 0.1 mmol), which was synthesized following the published procedure[1,2], and triphenylphosphine ligands (1.1 eq.) was dissolved in anhydrous tetrahydrofuran (2 ml). The reaction mixture was stirred in rt for 1 hour under N_2. After complete consumption of the starting molecules, the solvent and volatile was removed by vacuum at 50 ºC. The final products were recrystallized in suitable solvents to obtain **1-3** as pale-yellow or orange solids (90-95%).

1.3.1 Compound 1: ^{1}H NMR (400 MHz, $CDCl_3$) δ 7.94 (s, 1H), 7.76 – 7.69 (m, 6H), 7.61 (ddt, *J* = 6.8, 3.0, 1.5 Hz, 3H), 7.52 (ddd, *J* = 8.6, 5.2, 2.3 Hz, 6H), 7.18 (dd, *J* = 8.7, 1.7 Hz, 1H), 6.87 (dd, *J* = 8.6, 2.1 Hz, 1H), 6.37 (d, *J* = 2.1 Hz, 1H); ^{13}C NMR (101 MHz, $CDCl_3$) δ 161.9, 158.9, 157.2, 150.7, 132.7, 132.5, 132.4, 129.5, 129.2, 129.0, 128.6, 127.6, 123.5, 115.6, 108.8, 107.8, 107.6, 92.2; HRMS (ESI, M+H$^+$) calcd for $C_{28}H_{20}N_2O_2P$ 447.12624, found 447.12602.

1.3.2 Compound 2: ^{1}H NMR (400 MHz, $CDCl_3$) δ 8.00 (dd, *J* = 8.5, 2.6 Hz, 2H), 7.91 (ddd, *J* = 20.1, 8.9, 5.5 Hz, 5H), 7.80 – 7.73 (m, 4H), 7.64 (td, *J* = 7.2, 1.2 Hz, 2H), 7.58 – 7.51 (m, 7H), 7.19 (dd, *J* = 8.6, 1.4 Hz, 1H), 6.88 (dd, *J* = 8.6, 2.0 Hz, 1H), 6.44 (d, *J* = 1.9 Hz, 1H); ^{13}C NMR (101 MHz, $CDCl_3$) δ 161.6, 158.9, 157.2, 154.8, 152.4, 150.8, 133.5, 133.4, 132.9, 132.5, 132.4, 132.0, 130.9, 129.9, 129.6, 129.3, 129.2, 128.9, 128.5, 128.3, 127.3, 123.7, 123.5, 123.2, 123.1, 115.6, 108.9, 107.9, 107.8, 92.6; HRMS (ESI, $M+H^+$) calcd for $C_{34}H_{24}N_4O_2P$ 551.1636, found 551.1629.

1.3.3 Compound 3: ^{1}H NMR (400 MHz, $CDCl_3$) δ = 7.95 (s, 2H), 7.59 – 7.54 (m, 12H), 7.47 – 7.41 (m, 8H), 7.17 (dd, *J* = 8.8, 1.6 Hz, 2H), 6.76 (dd, *J* = 8.6, 1.7 Hz, 2H), 6.26 (d, *J* = 2.3 Hz, 2H), 4.54 – 4.51 (m, 4H), 4.16 – 4.14 (m, 4H). HRMS (ESI, $M+H^+$): cacld. For $C_{54}H_{36}FeN_4O_4P_2$: 922.6985 Da; Found: 922.6974 Da.

1.4 Spectroelectrochemical measurements and TCNQ analysis

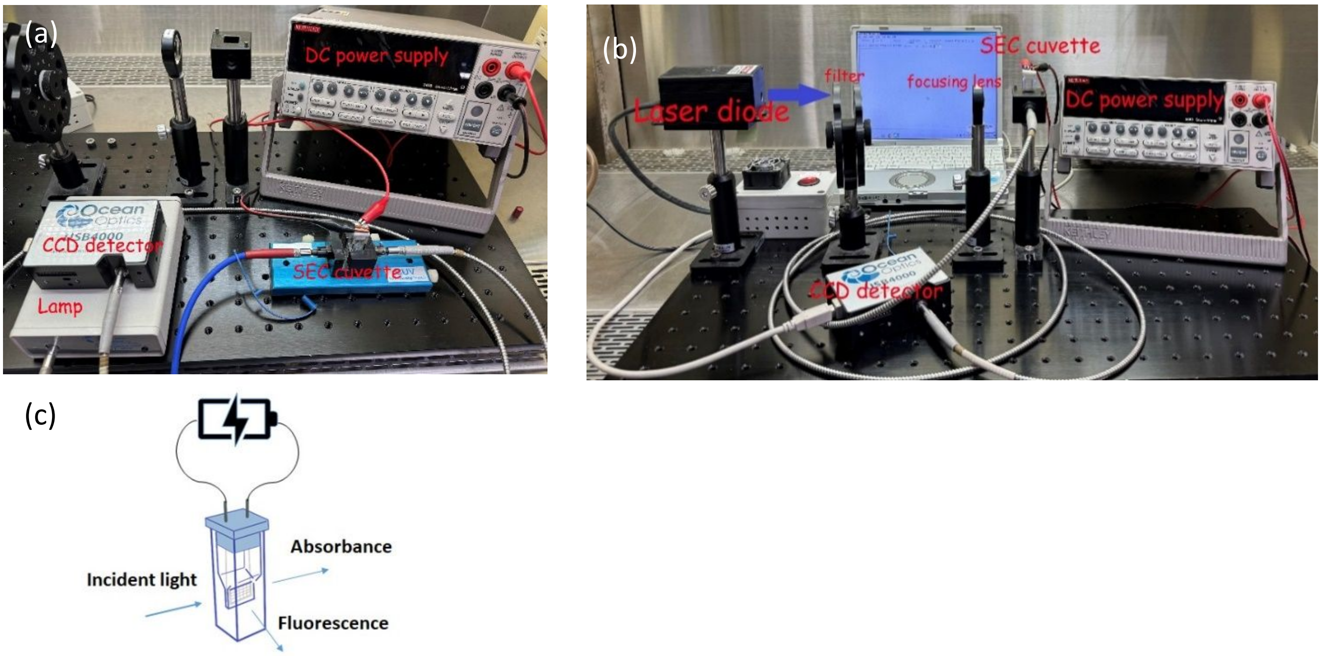

Figure S1. A home-built spectroelectrochemical (SEC) apparatus. (a) UV-vis absorption, (b) Fluorescence analysis. (c) A quartz SEC cuvette connected with a dry cell battery.

As shown in Figure S1, spectroelectrochemical (SEC) measurement was performed on a home-built apparatus equipped with either a deuterium tungsten halogen light source (DT-Mini, Ocean Optics) or 405-nm laser diode for absorption and fluorescence analysis, respectively; a CCD-array spectrometer (USB4000, Ocean Optics) was used as the detector for analyzing the fiber-based optical signal. The SEC set equipped with a specific cuvette of optical path length of 1.0 mm and a Pt gauze working electrode was purchased from ALS Co., Ltd. (Japan). Either a 3-volt battery or a DC power supply (E3631A, Agilent) were used as the power source to perform the electrochemical oxidation (ECO) process. The incident power of the commercially available laser diode was

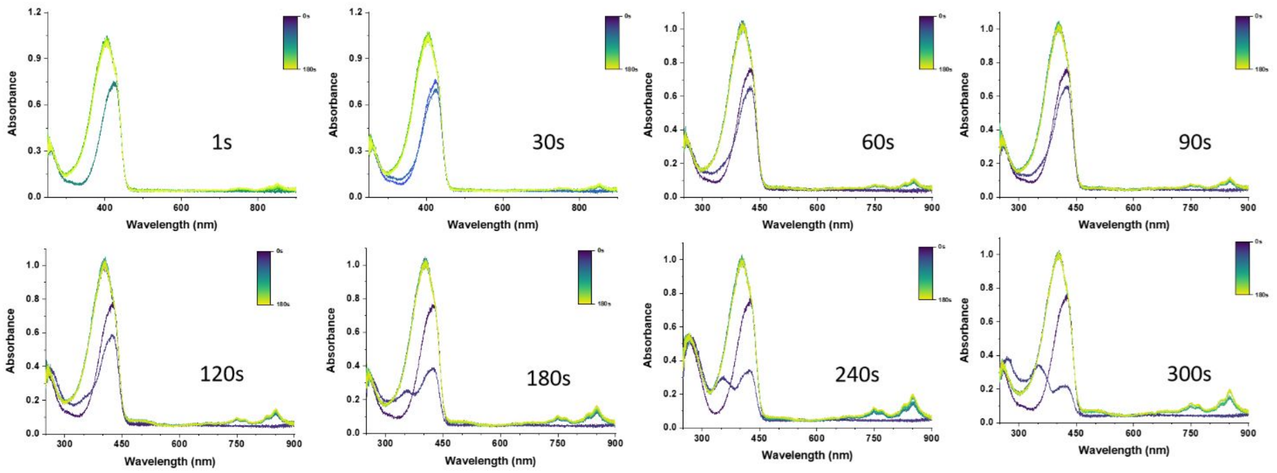

Figure S2. TCNQ analysis for THF solutions of compound **1** (1.25×10^{-4} M). The electrochemical oxidation time was set to 1-300 s, followed by TCNQ addition (1.4×10^{-3} M) and then monitoring for up to 180 s. The absorption spectra for each solution were recorded by bench type UV–Vis spectrometer. The ECO process was performed by a 3-volt battery.

carefully adjusted to record the fluorescence spectra with suitable intensity. During the ECO process, the lased power must be fixed, allowing us to real-time trace the change in fluorescence spectra. All the reactions were carried out in anhydrous THF because of its good solubility for coumarin-TPIPP compounds. The solutions were homogeneous and no precipitation was observed after the ECO treatment.

To confirm the radical formation after the ECO process, an excellent electron acceptor related to tetracyanoquinodimethane (TCNQ) was added to the ECO-treated solution of compound **1** to capture the formed radicals.[3] We analyzed the correlation between the ECO-treating time for compound **1** and generation of the radical anion of TCNQ. Before addition of the TCNQ, the oxidation time of compound **1** was set to be 1, 30, 60, 90, 120, 180, 240, and 300 s. It is found that the longer oxidation time resulted in more pronounced absorption peak at 350 nm before adding TCNQ, indicating that more oxidized **1** were formed, as shown in Figure S2. Subsequently, after the addition of TCNQ and monitoring for 180 s for each ECO-treated **1**, it is observable that the absorption peaks in near-infrared region (700-900 nm) progressively increased, indicating that the formation of radical anions and that longer oxidation time leads to more radical anions accumalted in the system.

1.5 Fluorescence life-time measurements

Fluorescence lifetimes were determined utilizing the Time-Correlated Single Photon Counting (TCSPC) technique. A digital converter (PicoQuant Time Harp 200), integrated into a computer system, measured the time delay between the excitation pulse and the detection of the first fluorescence photon. The fluorescence excitation source employed was a 375 nm pulsed diode laser emitting 80 ps pulses at a frequency of 40 MHz. These pulses were directed towards the sample

through a focused lens, and the resulting fluorescence light was collected by a set of collimated lenses. Subsequently, the fluorescence was detected by a single-photon counting photomultiplier tube (PMT 957, Hamamatsu), which was equipped with an Olympus HR320 spectrometer. The fitting method was employed using the built-in exponential decay equation within the Origin software. The fitting analysis of compound **1** was shown in Figure S3, suggesting increasing life-time from 2.54 to 3.75 ns after electrochemical oxidation.

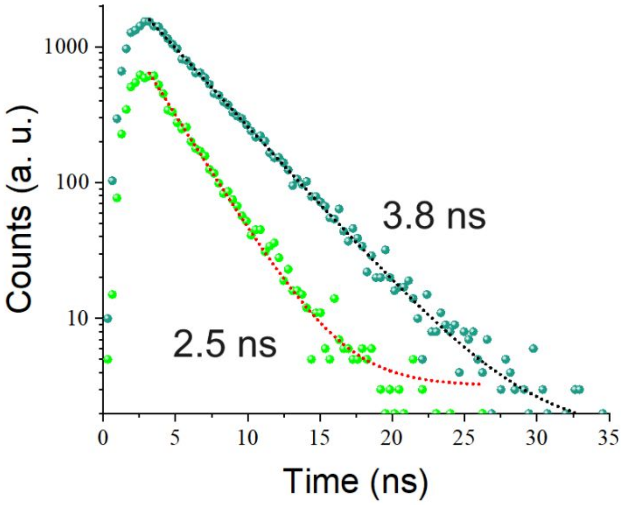

Figure S3. Fluorescence life-time analysis of compound **1**.

REFERENCES

[1] Hsia, L.-Y.; Chen, H.-N.; Chiang, C.-H.; Hung, M.-Y.; Wei, H.-K.; Luo, C.-W.; Kuo, M.-Y.; Luo, S.-Y.; Chu, C.-C. π-Extended Coumarins Derived with Nonhydrolyzable Iminophosphoranes as Two-Photon-Excited Fluorophores. *J. Org. Chem.* **2020**, *85* (14), 9361-9366.

[2] Lin, H.-Y.; Chen, C.-F.; Chen, C.-H.; Yeh, J.-L.; Huang, T.-T.; Chu, Y.-C.; Chu, C.-C. Polymer-Bound Coumarin–Triphenyliminophosphorane Derivatives as Turn-On Fluorescence Sensors for Water Content Detection via Non-Hydrolytic Staudinger Reaction. *ACS Appl. Polym. Mater.* **2022**, *4* (10), 7518-7527.

[3] Escobar, M.; Jin, Z.; Lucht, B. L. Electron-Donating Properties of p-Phenylene Phosphine Imides: An Electrochemical and Spectroscopic Investigation. *Org. Lett.* **2002**, *4* (13), 2213-2216.

	Parameter	Value
1	Origin	Varian
2	Spectrometer	mercury
3	Solvent	cdcl3
4	Temperature	25.0
5	Pulse Sequence	s2pul
6	Experiment	1D
7	Number of Scans	60
8	Receiver Gain	39
9	Relaxation Delay	1.0000
10	Acquisition Time	2.5575
11	Spectrometer Frequency	400.43
12	Spectral Width	6406.1
13	Lowest Frequency	-799.9
14	Nucleus	1H

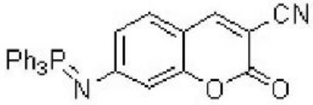

6a

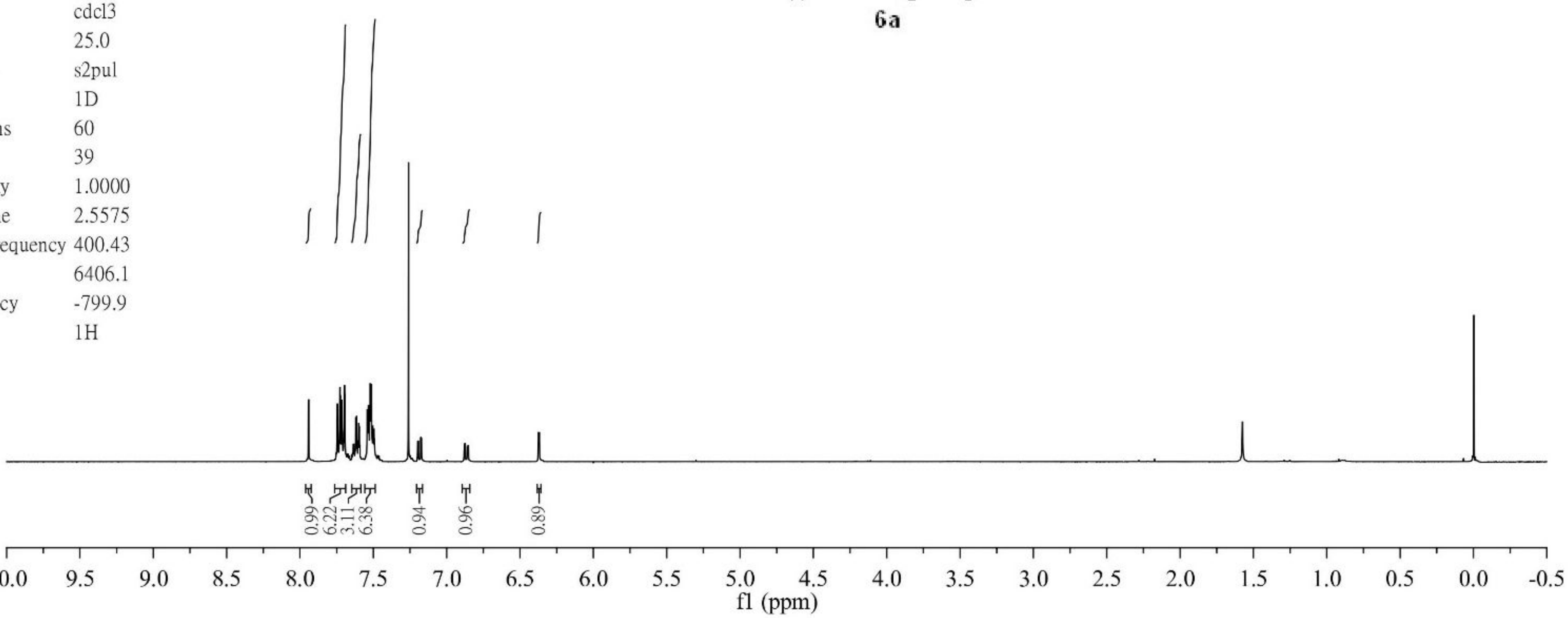

	Parameter	Value
1	Origin	Varian
2	Spectrometer	vnmrs
3	Solvent	d2o
4	Temperature	20.0
5	Pulse Sequence	s2pul
6	Experiment	1D
7	Number of Scans	164
8	Receiver Gain	30
9	Relaxation Delay	2.0000
10	Acquisition Time	1.3107
11	Spectrometer Frequency	100.53
12	Spectral Width	25000.0
13	Lowest Frequency	-1443.0
14	Nucleus	13C

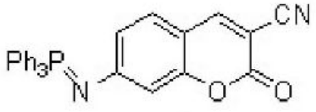

6a

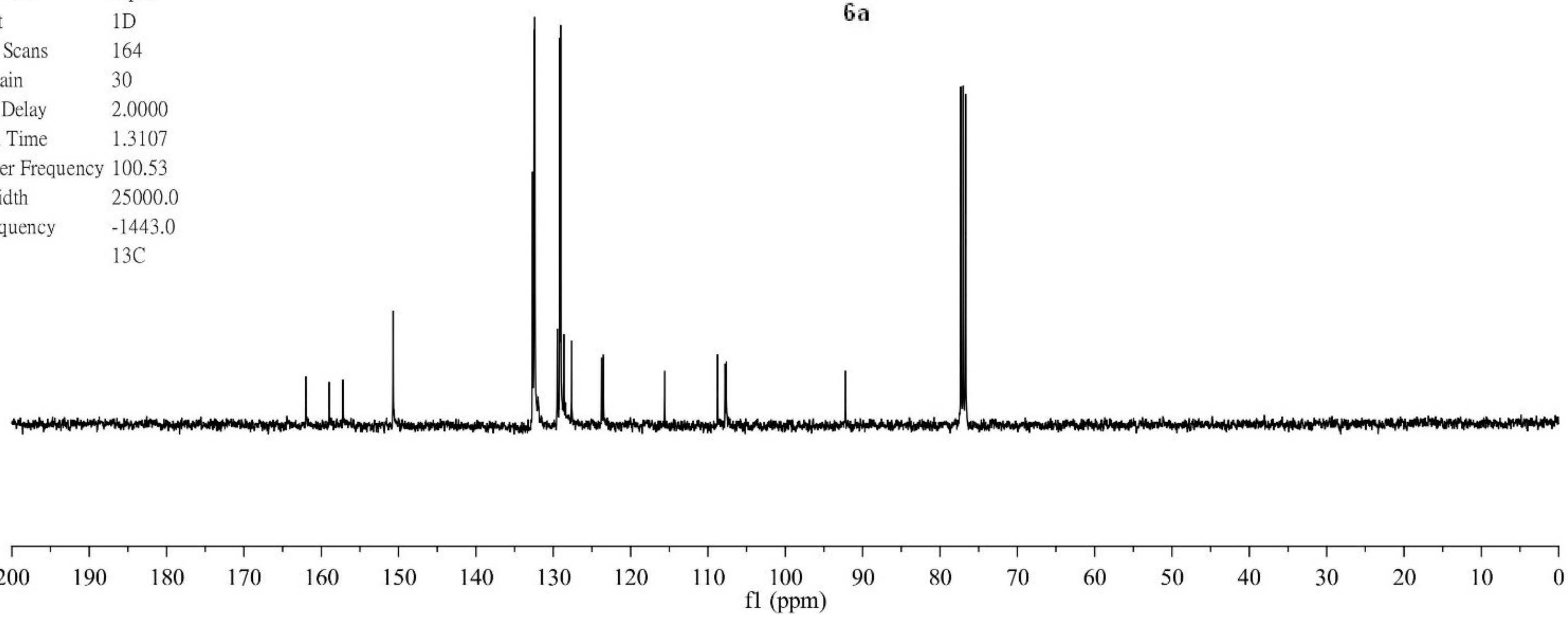

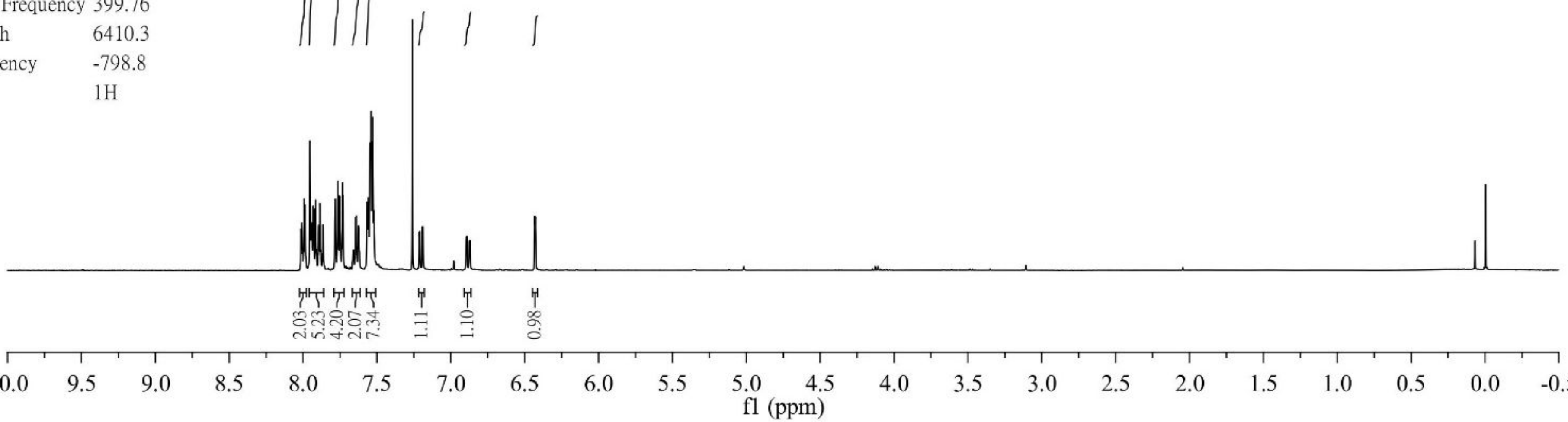

	Parameter	Value
1	Origin	Varian
2	Spectrometer	vnmrs
3	Solvent	cdcl3
4	Temperature	20.0
5	Pulse Sequence	s2pul
6	Experiment	1D
7	Number of Scans	24
8	Receiver Gain	48
9	Relaxation Delay	1.0000
10	Acquisition Time	2.5559
11	Spectrometer Frequency	399.76
12	Spectral Width	6410.3
13	Lowest Frequency	-798.8
14	Nucleus	1H

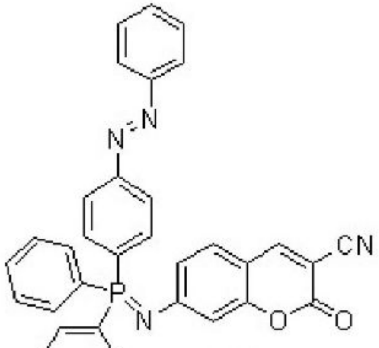

18

Parameter		Value
1	Origin	Varian
2	Spectrometer	vnmrs
3	Solvent	cdcl3
4	Temperature	20.0
5	Pulse Sequence	s2pul
6	Experiment	1D
7	Number of Scans	172
8	Receiver Gain	30
9	Relaxation Delay	2.0000
10	Acquisition Time	1.3107
11	Spectrometer Frequency	100.53
12	Spectral Width	25000.0
13	Lowest Frequency	-1447.1
14	Nucleus	13C

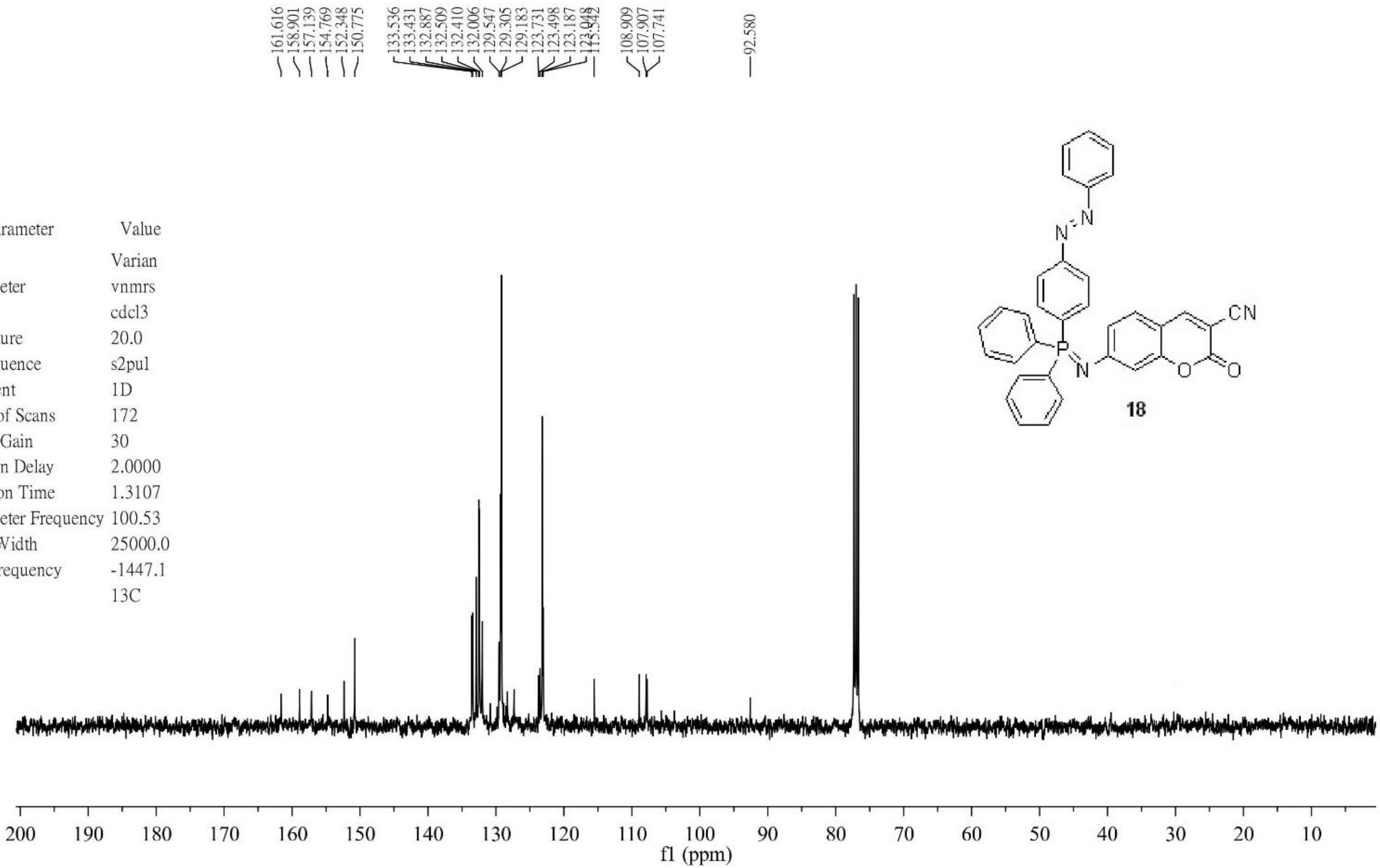

18

Parameter	Value
Origin	JEOL
Spectrometer	NCHU
Solvent	CDCl3
Temperature	
Pulse Sequence	
Experiment	1D
Number of Scans	8
Receiver Gain	
Relaxation Delay	2.0000
Acquisition Time	2.4429
Spectrometer Frequency	399.78
Spectral Width	9005.8
Lowest Frequency	-2104.2
Nucleus	Proton

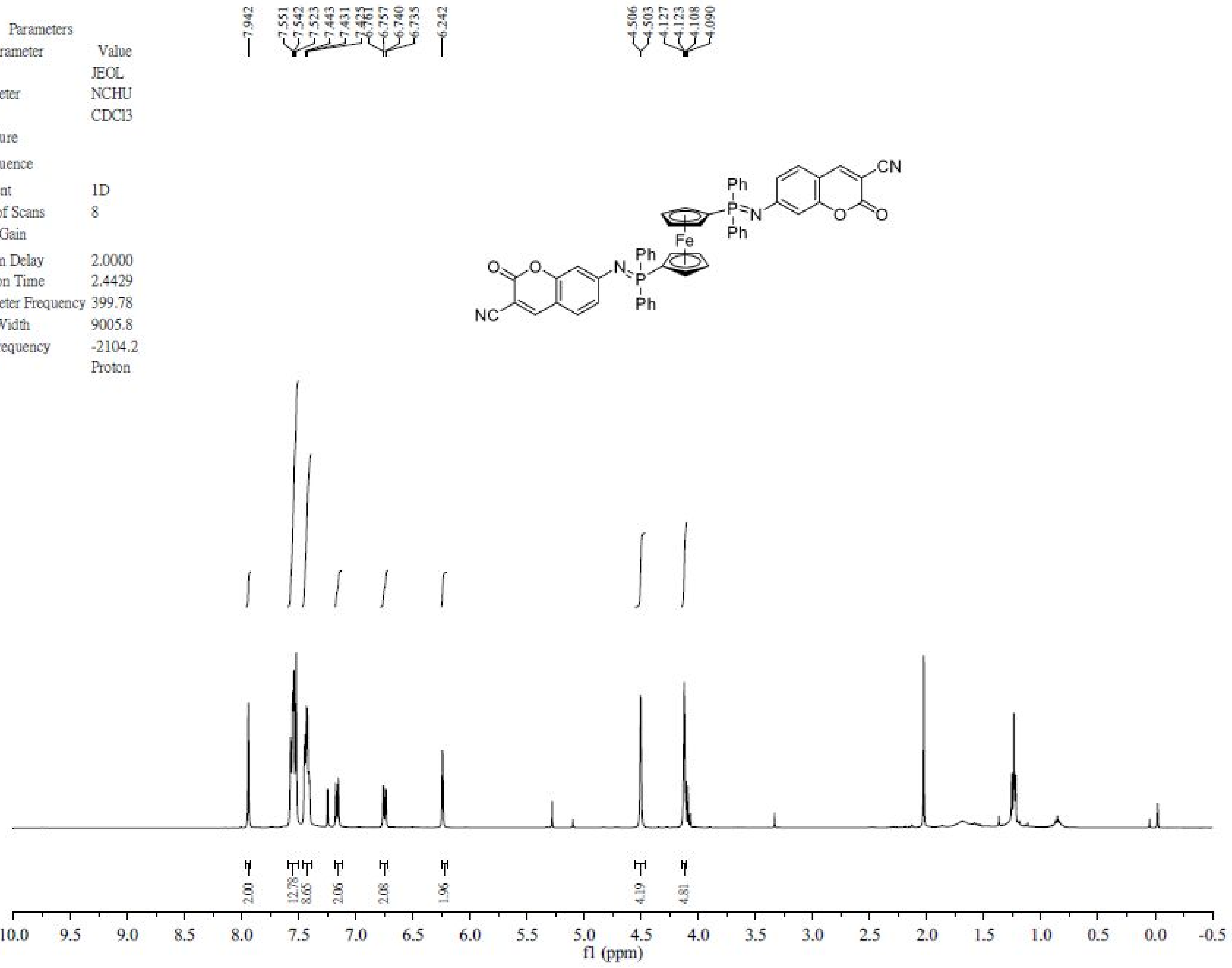

Supplement: Supplementary file 1 — pg4c00082_si_001.pdf [file pg4c00082_si_001.pdf]
